# Changes by Era in Risk Factors and Outcomes Among Deceased Donor Kidney Transplant Recipients With Delayed Graft Function

**DOI:** 10.1111/ctr.70484

**Published:** 2026-02-15

**Authors:** Camille C. Ylagan, Paul E. Schindler, Dave B. Patel, Carrie Thiessen, Adam P. Bregman, Didier Mandelbrot, Brad C. Astor, Sandesh Parajuli

**Affiliations:** ^1^ Division of Nephrology Department of Medicine University of Wisconsin School of Medicine and Public Health Madison Wisconsin USA; ^2^ Division of Transplant Surgery Department of Surgery University of Wisconsin School of Medicine and Public Health Madison Wisconsin USA; ^3^ Department of Population Health Sciences University of Wisconsin School of Medicine and Public Health Madison Wisconsin USA

**Keywords:** deceased donor, DGF, kidney transplant, outcomes, recent era

## Abstract

**Introduction:**

There are no effective therapeutic agents for preventing or treating delayed graft function (DGF) among deceased donor kidney transplant recipients (DDKTRs). Donor and recipient factors are important to predicting DGF and associated outcomes, which we hypothesize differed over time.

**Methods:**

DDKTRs were stratified by transplant year into four eras—E1 (2000–2005), E2 (2006–2011), E3 (2012–2017), and E4 (2018–2021). We analyzed risk factors for DGF, along with one‐year uncensored graft failure (UCGF), death‐censored graft failure (DCGF), death with a functioning graft (DWFG), and acute rejection (AR) by era.

**Results:**

A total of 3085 DDKTRs were included (E1: 804, E2: 882, E3: 909, E4: 490). The proportion of patients with DGF differed significantly by era. Duration of DGF and median dialysis count were lower in recent eras.

In E1‐E4, donation after circulatory death, higher donor terminal serum creatinine, and pretransplant duration of dialysis were risk factors for DGF, while preemptive transplant was associated with lower odds of DGF. Other factors were not consistently associated with DGF across eras.

The risk of one‐year AR was significantly lower in E3 (aHR: 0.46; 95% CI: 0.30–0.69, *p* < 0.001) and E4 (aHR: 0.16; 95% CI: 0.07–0.36, *p* < 0.001) compared to E1. There were trends towards decreased risk for UCGF and DWFG in E2, E3, and E4.

**Conclusion:**

Some risk factors for DGF remained consistent, while others differed. Likely due to improved management, the risk for AR in the DGF setting improved in recent eras. There were trends of improved uncensored graft and patient survival in recent eras.

**Abbreviations**
aHR: adjusted hazard ratio
AKI: acute kidney injury
AMR: antibody‐mediated rejection
aOR: adjusted odds ratio
AR: acute rejection
BMI: body mass index
CI: confidence interval
CIT: cold ischemia time
CNI: calcineurin inhibitors
cPRA: calculated panel reactive antibody
DBD: donation after brain death
DCD: donation after circulatory death
DCGF: death‐censored graft failure
DDKTR: deceased donor kidney transplant recipient
DGF: delayed graft function
DWFG: death with a functioning graft
eGFR: estimated glomerular filtration rate
ESKD: end‐stage kidney disease
HLA: human leukocyte antigen
IRI: ischemia‐reperfusion injury
IVIG: intravenous immunoglobulin
KDIGOKidney Disease: Kidney DiseaseImproving Global Outcomes
KDPI: kidney donor profile index
KTR: kidney transplant recipient
TCMR: T‐cell‐mediated rejection
UCGF: uncensored graft failure
uHR: unadjusted hazard ratio
UNOS/OPTN: United Network for Organ Sharing/Organ Procurement and Transplantation Network
WIT: warm ischemia time

## 1 Introduction

Kidney transplants are lifesaving procedures for patients with end‐stage kidney disease (ESKD) [1]. The increased demand for kidney transplants and the shortage of available kidneys have motivated efforts to safely expand the kidney donor pool. For example, there has been increased use of kidneys from donation after circulatory death (DCD) donors and from donors aged 50 years and older [2, 3]. Additionally, there has been increased regional and national allocation of kidneys, resulting in longer cold ischemia times (CIT) [4].

Delayed graft function (DGF) is a posttransplant acute kidney injury (AKI), commonly characterized by the use of dialysis within seven days of transplant, although more than 15 definitions of DGF have been published [5]. One study reported an incidence of DGF of 23% between 1998 and 2004 and another cited an incidence of DGF of 29% between 2010 and September 2018 [6, 7]. There have been numerous studies on risk factors for DGF and outcomes following DGF. Risk factors for DGF include DCD, increasing donor age, CIT, donor creatinine, and recipient body mass index (BMI) [8]. DGF is associated with increased risk of acute rejection (AR) [9], increased risk of graft failure [8], and decreased patient survival [6]. DGF is also associated with prolonged length of stays and financial costs to the healthcare system [10]. However, research on how such risk factors and outcomes have changed over time is limited. Furthering the understanding of patterns in risk factors and outcomes over time is essential for preventing and treating DGF.

Given the changing donor pool, examining proper donor and recipient factors is vital to prevent these detrimental outcomes. Additionally, with the advancements in immunosuppression and management for DGF, we sought to determine if there have been changes in DGF‐related outcomes over time. We hypothesize that there have been changes in risk factors for and outcomes of DGF in recent eras versus previous eras.

## 2 Methods

### 2.1 Study Population and Design

This was a single‐center observational study of all adult kidney transplant recipients (KTRs) who were transplanted at the University of Wisconsin‐Madison between January 1, 2000 and December 31, 2021. Recipients eligible for inclusion were adult deceased donor kidney‐only transplant recipients (DDKTRs). We excluded all living‐donor KTRs, along with those with graft failure within seven days posttransplant. DDKTRs were stratified by transplant year into four eras – E1 (2000–2005), E2 (2006–2011), E3 (2012–2017), and E4 (2018–2021). E1 was used as the reference group for statistical analysis. We categorized patients into these four eras to divide eras into six years each (except for E4) and to investigate outcomes before and after DGF Clinic was implemented in 2011 (see below).

Primary outcomes of interest included duration of DGF, number of dialysis sessions needed, uncensored graft failure (UCGF), death‐censored graft failure (DCGF), death with a functioning graft (DWFG), and AR within 12 months posttransplant.

This study was approved by the University of Wisconsin Institutional Review Board (IRB protocol number: 2014‐1072). This study adhered to the Declaration of Helsinki. The clinical and research activities reported were consistent with the Principles of the Declaration of Istanbul on Organ Trafficking and Transplant Tourism. Due to the nature of this study, informed consent explicit to this research was not obtained from patients.

### 2.2 Variables and Definitions

Although numerous definitions of DGF exist in the literature, the most commonly used definition and the one used in our study is the need for dialysis within seven days of the kidney transplant [5]. At our university, the definition of DGF has been consistent throughout the study period. UCGF was defined as graft failure resulting from any cause, including death. DCGF was defined as graft failure prior to death in which the patient required chronic dialysis or repeat transplantation. DWFG was defined as a patient dying with a functioning graft. All AR was biopsy‐proven based on the then‐Banff criteria. Duration of DGF was defined as the duration between transplant and last dialysis posttransplant.

Recipient age, sex, race (White vs. non‐White), BMI, cause of ESKD (diabetes, hypertension, glomerulonephritis, polycystic kidney disease), induction immunosuppression (alemtuzumab, anti‐thymocyte globulin, basiliximab/daclizumab), preemptive transplant proportion, and pretransplant duration of dialysis were obtained. Immunological factors, specifically calculated panel reactive antibody (cPRA) > 80%, human leukocyte antigen (HLA) mismatch per 1, and previous transplant, also were collected. Donor factors gathered were age, sex, race (White vs. non‐White), BMI, cardiovascular cause of death, total serum creatinine, kidney donor profile index (KDPI), right kidney, CIT, hypothermic machine perfusion, and DCD versus donation after brain death (DBD). All data were obtained from the UW transplant database, except for the hypothermic machine perfusion or static cold storage, which was obtained from the Organ Procurement and Transplantation Network Standard Transplant Analysis and Research.

### 2.3 DGF Management

Most DDKTRs who received a transplant prior to 2011 and experienced DGF were treated as inpatients. Since July 2011, KTRs with DGF were treated as outpatients in a multidisciplinary DGF clinic [11]. Patients with DGF were either discharged from the University of Wisconsin Hospital and Clinics to their home or to a nearby hotel with a support person (if their home was greater than 40 miles away). The patient was seen by the transplant providers within 1–3 days of discharge. Many patients required follow‐up in the DGF clinic 3 days per week. Patients had lab work done prior to each clinic visit. Patients received dialysis in the inpatient dialysis unit if indicated. Since the implantation of the DGF clinic in 2011, there has been a significant decrease in length of stay, with a mean of 10.9 days among patients with DGF pre‐DGF clinic compared to 6.1 days post‐DGF clinic (*p* < 0.001) [11]. There was no significant difference in the rate of 30‐day admission (16% vs. 21 % *p* = 0.39). Also, the rate of AR was significantly lower post‐DGF clinic [11].

### 2.4 Immunosuppressive Protocols

Most KTRs at our institution received induction with either a depleting agent (anti‐thymocyte globulin or alemtuzumab) or a nondepleting agent (basiliximab) based on immunological risk factors. This treatment was followed by immunosuppressive therapy with tacrolimus, mycophenolic acid, with or without prednisone [12, 13]. Patients with pretransplant donor‐specific antibodies, patients with glomerulonephritis as the cause of kidney disease, and patients planned for early steroid withdrawal were more likely to receive a depleting agent for induction [14]. Patients at risk for DGF generally received a depleting agent, per the transplant surgeon's decision.

### 2.5 Kidney Allograft Biopsy

Biopsies were mainly performed when there was an unexplained rise in serum creatinine or proteinuria. Additionally, protocol biopsies were performed at 3 and 12 months for individuals with pretransplant donor‐specific antibodies or patients who developed *de novo* donor‐specific antibodies [14]. Among recipients with DGF, graft biopsy was performed around 7–14 days posttransplant if there was no significant improvement in the graft function.

### 2.6 Rejection Protocols

Treatment for allograft rejection varied based on the severity and timing of antibody‐mediated rejection (AMR) and acute T‐cell‐mediated rejection (TCMR) [13, 15]. AMR within 3 months posttransplant was treated with dexamethasone bolus, plasmapheresis, and intravenous immunoglobulin (IVIG). AMR occurring more than 3 months posttransplant included a dexamethasone bolus with taper, IVIG, plus/minus rituximab depending on rejection severity. TCMR was treated with a steroid pulse for borderline and Banff stage I rejection. A steroid pulse and anti‐thymocyte globulin were used to treat Banff stages II and III.

### 2.7 Statistical Analysis

Continuous data were analyzed using Student's t‐test or the Wilcoxon rank‐sum test, as appropriate. Categorial data were compared using Fisher's exact test or chi‐square test. *p* values ≤ 0.05 were considered statistically significant. Multivariate logistic regression models were used to calculate adjusted odds ratios for DGF for risk factors in each era. Bivariate and multivariate Cox proportional hazards models were used to calculate hazard ratios for the first posttransplant year by era for the outcomes of UCGF, DCGF, DWFG, and AR. All variables from the baseline characteristics were included in the multivariate analysis except for the machine perfusion. Perfusion history was not included, as it was not from the UW data, and also we did not have other details of perfusion including time and pressure. Follow‐up was truncated at 12 months. Follow‐up was between one week to one year after transplant. Kaplan–Meier analyses were performed for these outcomes by era.

## 3 Results

A total of 3085 DDKTRs were included in this study (E1: 804, E2: 882, E3: 909, E4: 490). The proportion of DDKTRs with DGF significantly differed by era (E1: 25.8%, E2: 26.6%, E3: 32.7%, E4: 26.7%, *p* = 0.005). The median duration of DGF decreased in recent eras. In E1, the proportion of patients with a median duration of DGF between 7–13 days was highest (38.7%), followed by ≥14 days (34.8%), and then between 1–6 days (26.6%). In contrast, in E3, the proportion of patients with a median duration of DGF between 1–6 days was highest (46.1%), followed by between 7–13 days (30.0%), and then ≥14 days (23.9%). Similarly, in E4, the proportion of patients with a median duration of DGF between 1–6 days was highest (40.5%), followed by between 7–13 days (35.9%), and then ≥14 days (23.7%). Also, the median number of dialysis sessions needed was lower in the recent eras. Specifically, the median number of dialysis sessions needed was 4 sessions (IQR: 2–7) in E1, 4 sessions (IQR: 2–6) in E2, 3 sessions (IQR: 2–5) in E3, and 3 sessions (IQR: 2–5) in E4, p‐trend = 0.008. Graft function, measured by estimated glomerular filtration rate (eGFR) at 6 ± 1 months, differed significantly across eras (E1: 65.0 ± 25.5, E2: 70.1 ± 26.7, E3: 70.2 ± 26.8, E4: 68.2 ± 28.6, *p* = 0.007).

In recent eras, DDKTRs were older (E1: 51.0 ± 12.4 years, E2: 52.1 ± 12.6 years, E3: 52.9 ± 12.4 years, E4: 54.2 ± 12.7 years, *p* = 0.008) and had higher mean BMI (E1: 27.1 ± 5.5 kg/m^2^, E2: 27.8 ± 5.0 kg/m^2^, E3: 28.3 ± 5.3 kg/m^2^, E4: 28.9 ± 5.4 kg/m^2^, *p* = 0.010) compared to in previous eras. There was a decrease in the proportion of individuals who received a previous transplant in recent eras (E1: 23.0%, E2: 21.2%, E3: 20.6%, E4: 14.9%, *p* = 0.005). The proportion of non‐White DDKTRs, recipient cause of ESKD, recipient induction immunosuppression, pretransplant duration of dialysis, cPRA > 80%, and degree of HLA mismatch also differed significantly across eras (Table 1).

**TABLE 1:** Baseline Characteristics.

		2000–2005	2006–2011	2012–2017	2018–2021	P
Total number of transplant		804	882	909	490	—
Recipients factors	Mean age (years)	51.0 (12.4)	52.1 (12.6)	52.9 (12.4)	54.2 (12.7)	**0.008**
	Female (%)	39.6	39.0	38.1	40.4	0.840
	Non‐White (%)	19.8	54.9	60.5	34.1	**<0.001**
	Mean body mass index (kg/m^2^)	27.1 (5.5)	27.8 (5.0)	28.3 (5.3)	28.9 (5.4)	**0.010**
	Causes of ESKD (%)					
	Diabetes	26.2	25.9	26.6	30.2	**0.001**
	Hypertension	12.6	12.4	16.6	16.1	
	Glomerulonephritis	23.1	25.6	25.1	24.5	
	Polycystic kidney disease	11.1	13.4	9.9	12.9	
	Other	27.0	22.8	21.8	16.3	
	Induction immunosuppression (%)					
	Alemtuzumab	49.9	1.5	7.6	16.1	**<0.001**
	Anti‐thymocyte globulin	12.8	18.4	34.9	64.3	
	Basiliximab/Daclizumab	37.3	80.2	57.5	19.6	
	Preemptive transplant (%)	13.3	14.2	11.7	10.4	0.160
	Pretransplant duration, median (IQR)	23 (9, 43)	22 (9, 39)	34 (16, 58)	40 (22, 61)	**0.040**
Immunology factors	cPRA > 80%	17.5	8.7	19.0	11.0	**<0.001**
	Mean HLA mismatch (per 1)	3.5 (1.9)	4.0 (1.6)	4.0 (1.5)	4.1 (1.4)	**0.020**
	Previous transplant (%)	23.0	21.2	20.6	14.9	**0.005**
Donor factors	Mean age (years)	43.0 (17.3)	43.4 (15.6)	41.5 (16.1)	42.7 (15.5)	**0.002**
	Female (%)	40.6	35.7	37.3	38.2	0.230
	Non‐White (%)	8.1	6.8	8.5	11.4	**0.030**
	Mean body mass index (kg/m^2^)	27.3 (7.1)	28.5 (7.0)	29.0 (8.2)	29.5 (7.5)	**0.010**
	Cause of death: Cardiovascular (%)	42.3	32.0	27.9	23.5	**<0.001**
	Terminal serum Creatinine (mg/dl)	1.14 (1.25)	0.96 (0.52)	0.92 (0.47)	1.00 (0.57)	**0.010**
	Mean kidney donor profile index (%)	51.6 (22.5)	48.6 (21.1)	46.8 (19.9)	48.9 (23.0)	**0.007**
	Right kidney (%)	56.0	54.2	52.3	50.0	0.130
	Cold ischemia time (h)					
	<12 h	5.7	33.7	28.6	36.2	**<0.001**
	12–18 h	26.5	37.4	33.4	30.1	
	19–24 h	40.7	22.3	27.7	24.3	
	>24 h	27.1	6.6	10.2	9.4	
	Machine perfusion (%) Static cold storage/unknown (%)	719 (89) 85 (11)	773 (88) 109 (12)	647 (71) 262 (29)	351(72) 139 (28)	**<0.001**
	Deceased donor (%) DBD	81.6	68.1	69.5	64.5	**<0.001**
DGF‐related factors	DGF patients (%)	25.8	26.6	32.7	26.7	**0.005**
	Median duration of DGF, from transplant to last dialysis date (%)					
	1–6 days	26.6	32.3	46.1	40.5	**<0.001**
	7–13 days	38.7	39.6	30.0	35.9	
	≥14 days	34.8	28.1	23.9	23.7	
	Number of dialysis sessions (median, IQR)	4 (2, 7)	4 (2, 6)	3 (2, 5)	3 (2, 5)	**0.008**
Graft function	eGFR 6 ± 1 months	65.0 (25.5)	70.1 (26.7)	70.2 (26.8)	68.2 (28.6)	**0.007**

cPRA: calculated panel reactive antibody; DBD: donation after brain death; DGF: delayed graft function; eGFR: estimated glomerular filtration rate; ESKD: end‐stage kidney disease; HLA: human leukocyte antigen; IQR: interquartile range.

Additionally, the mean donor BMI increased significantly over time (E1: 27.3 ± 7.1 kg/m^2^, E2: 28.5 ± 7.0 kg/m^2^, E3: 29.0 ± 8.2 kg/m^2^, E4: 29.5 ± 7.5 kg/m^2^, *p* = 0.010). There was also a decreasing proportion of donors with a cardiovascular cause of death over time (E1: 42.3%, E2: 32.0%, E3: 27.9%, E4: 23.5%, *p* < 0.001). The mean donor age, proportion of non‐White donors, donor terminal serum creatinine, mean KDPI, cold ischemia time, and proportion of DBD donors also differed significantly across eras (Table 1).

### 3.1 Risk Factors by Era

DCD, higher donor terminal serum creatinine, pretransplant duration of dialysis, and preemptive transplant were consistent factors across eras. DCD was a risk factor for DGF in E1 (aOR: 3.61, 95% CI: 2.33–5.62, *p* < 0.001), E2 (aOR: 4.28, 95% CI: 2.90–6.31, *p* < 0.001), E3 (aOR: 5.19, 95% CI: 3.62–7.45, *p* < 0.001), and E4 (aOR: 2.63, 95% CI: 1.58–4.39, *p* < 0.001) (Table 2). This remained the case after excluding preemptive transplants from analysis: E1 (aOR: 3.80, 95% CI: 2.42–5.98, *p* < 0.001), E2 (aOR: 4.38, 95% CI: 2.94–6.55, *p* < 0.001), E3 (aOR: 5.37, 95% CI: 3.71–7.78, *p* < 0.001), and E4 (aOR: 2.87, 95% CI: 1.70–4.86, *p* < 0.001) (Supplemental Table 1). Donor terminal serum creatinine was also a risk factor for DGF in E1 (aOR: 1.23, 95% CI: 1.02–1.50, *p* = 0.032), E2 (aOR: 1.75, 95% CI: 1.25–2.46, *p* = 0.001), E3 (aOR: 2.51, 95% CI: 1.72–3.65, *p* < 0.001), and E4 (aOR: 1.83, 95% CI: 1.21–2.75, *p* = 0.004) (Table 2). This remained after excluding preemptive transplants from analysis: E1 (aOR: 1.22, 95% CI: 1.01–1.46, *p* = 0.035), E2 (aOR: 1.84, 95% CI: 1.30–2.61, *p* = 0.001), E3 (aOR: 2.58, 95% CI: 1.76–3.77, *p* < 0.001), and E4 (aOR: 1.91, 95% CI: 1.26–2.90, *p* = 0.002) (Supplemental Table 1). Pretransplant duration of dialysis was also a risk factor for DGF in E1 (aOR: 1.25, 95% CI: 1.15–1.35, *p* < 0.001), E2 (aOR: 1.29, 95% CI: 1.17–1.42, *p* < 0.001), E3 (aOR: 1.08, 95% CI: 1.02–1.15, *p* = 0.007), and E4 (aOR: 1.13, 95% CI: 1.02–1.25, *p* = 0.020). In contrast, preemptive transplant was associated with lower odds of DGF in all eras: E1 (aOR: 0.15, 95% CI: 0.06–0.36, *p* < 0.001), E2 (aOR: 0.16, 95% CI: 0.07–0.35, *p* < 0.001), E3 (aOR: 0.10, 95% CI: 0.04–0.23, *p* < 0.001), and E4 (aOR: 0.12, 95% CI: 0.03–0.44, *p* = 0.001) (Table 2).

**TABLE 2:** Risk factors for DGF for deceased donor kidney transplant patients by era.

		2000–2005	2006–2011	2012–2017	2018–2021
		aOR (95% CI; P)	aOR (95% CI; P)	aOR (95% CI; P)	aOR (95% CI; P)
Recipients factors	Age (per year)	0.93 (0.79, 1.11), 0.421	0.89 (0.76, 1.03), 0.127	0.99 (0.86, 1.15), 0.937	1.25 (1.00, 1.57), 0.054
	Female	0.82 (0.56, 1.19), 0.297	0.81 (0.57, 1.16), 0.253	0.70 (0.49, 0.98), **0.040**	0.53 (0.32, 0.89), **0.017**
	Non‐White	1.37 (0.85, 2.21), 0.192	2.03 (1.34, 3.07), **0.001**	0.86 (0.59, 1.25), 0.426	1.13 (0.66, 1.95), 0.649
	Body mass index (per Kg/m2)	1.09 (1.05, 1.13), **<0.001**	1.08 (1.04, 1.12), **<0.001**	1.06 (1.03, 1.10), **<0.001**	1.04 (0.99, 1.09), 0.085
	Causes of ESKD (%)				
	Diabetes	Ref	Ref	Ref	Ref
	Hypertension	0.55 (0.30, 1.01), 0.052	0.74 (0.42, 1.32), 0.312	1.03 (0.63, 1.69), 0.908	1.14 (0.57, 2.24), 0.715
	Glomerulonephritis	0.53 (0.32, 0.89), **0.016**	0.66 (0.40, 1.07), 0.092	0.94 (0.58, 1.52), 0.801	0.83 (0.41, 1.67), 0.596
	Polycystic kidney disease	0.61 (0.31, 1.18), 0.141	0.74 (0.41, 1.35), 0.328	0.67 (0.34, 1.33), 0.256	0.96 (0.42, 2.19), 0.921
	Other	0.63 (0.38, 1.03), 0.067	0.85 (0.51, 1.40), 0.515	1.00 (0.61, 1.64), 0.996	0.85 (0.41, 1.79), 0.675
	Induction immunosuppression				
	Alemtuzumab	Ref	Ref	Ref	Ref
	Anti‐thymocyte globulin	1.76 (0.97, 3.19), 0.061	1.24 (0.34, 4.51), 0.745	0.61 (0.33, 1.13), 0.115	0.37 (0.20, 0.71), **0.003**
	Basiliximab/Daclizumab	1.37 (0.90, 2.07), 0.140	0.83 (0.24, 2.94), 0.778	0.44 (0.24, 0.79), **0.007**	0.19 (0.08, 0.43), **<0.001**
	Preemptive transplant	0.15 (0.06, 0.36), **<0.001**	0.16 (0.07, 0.35), **<0.001**	0.10 (0.04, 0.23), **<0.001**	0.12 (0.03, 0.44), **0.001**
	Pretransplant duration of dialysis (per year)	1.25 (1.15, 1.35); **<0.001**	1.29 (1.17, 1.42); **<0.001**	1.08 (1.02, 1.15); **0.007**	1.13 (1.02, 1.25); **0.020**
Immunologic factors	HLA mismatch (per 1)	1.04 (0.93, 1.16), 0.468	0.96 (0.86, 1.08), 0.548	1.09 (0.97, 1.22), 0.141	0.92 (0.77, 1.09), 0.312
	Previous transplant	1.49 (0.91, 2.44), 0.113	1.00 (0.63, 1.60), 0.999	1.36 (0.86, 2.14), 0.185	1.41 (0.69, 2.86), 0.343
Donor factors	Age (per year)	1.09 (0.92, 1.29), 0.319	1.31 (1.09, 1.58), **0.003**	1.21 (1.04, 1.40), **0.014**	1.18 (0.93, 1.49), 0.176
	Female	1.12 (0.77, 1.63), 0.553	0.84 (0.57, 1.22), 0.353	0.99 (0.70, 1.41), 0.964	0.55 (0.33, 0.91), **0.021**
	Non‐White	0.83 (0.41, 1.70), 0.619	1.49 (0.71, 3.13), 0.290	0.62 (0.32, 1.20), 0.155	1.00 (0.45, 2.23), 0.992
	Body mass index (per kg/m2)	1.04 (1.01, 1.06), **0.009**	1.03 (1.01, 1.06), **0.011**	1.01 (0.99, 1.03), 0.592	1.00 (0.97, 1.03), 0.926
	Cause of death: cardiovascular	1.34 (0.88, 2.03), 0.168	1.42 (0.95, 2.12), 0.089	0.90 (0.60, 1.35), 0.621	1.21 (0.68, 2.17), 0.511
	DCD	3.61 (2.33, 5.62), **<0.001**	4.28 (2.90, 6.31), **<0.001**	5.19 (3.62, 7.45), **<0.001**	2.63 (1.58, 4.39), **<0.001**
	Terminal serum creatinine (mg/dl)	1.23 (1.02, 1.50), **0.032**	1.75 (1.25, 2.46), **0.001**	2.51 (1.72, 3.65), **<0.001**	1.83 (1.21, 2.75), **0.004**
	Kidney donor profile index	1.00 (0.99, 1.02), 0.394	1.00 (0.99, 1.01), 0.993	1.01 (1.00, 1.02), 0.059	1.01 (0.99, 1.03), 0.207
	Right kidney	0.95 (0.66, 1.37), 0.800	1.10 (0.77, 1.56), 0.607	1.54 (1.11, 2.14), **0.009**	1.32 (0.82, 2.11), 0.251
	Cold ischemia time				
	<12 h	Ref	Ref	Ref	Ref
	12–18 h	1.28 (0.54, 3.02), 0.576	0.94 (0.63, 1.40), 0.765	0.91 (0.60, 1.39), 0.675	0.99 (0.56, 1.74), 0.969
	19–24 h	1.49 (0.65, 3.40), 0.347	1.33 (0.82, 2.18), 0.249	1.15 (0.75, 1.77), 0.526	0.99 (0.53, 1.85), 0.982
	>24 h	1.95 (0.83, 4.60), 0.126	0.88 (0.39, 1.97), 0.747	1.62 (0.90, 2.93), 0.109	1.17 (0.50, 2.72), 0.717

* All variables were used for adjusted models.

aOR: adjusted odds ratio; CI: confidence interval; cPRA: calculated panel reactive antibody.

DCD: donation after circulatory death; DGF: delayed graft function; ESKD: end‐stage kidney disease.

HLA: human leukocyte antigen; Ref: reference.

Other factors, such as higher recipient BMI, higher donor BMI, and induction immunosuppression, were not consistently associated with DGF across eras. More specifically, higher recipient BMI was a risk factor for DGF in E1 (aOR: 1.09, 95% CI: 1.05–1.13, *p* < 0.001), E2 (aOR: 1.08, 95% CI: 1.04–1.12, *p* < 0.001), and E3 (aOR: 1.06, 95% CI: 1.03–1.10, *p* < 0.001) but not in E4 (aOR: 1.04, 95% CI: 0.99–1.09, *p* = 0.085). Higher donor BMI was a risk factor for DGF in E1 (aOR: 1.04, 95% CI: 1.01–1.06, *p* = 0.009) and E2 (aOR: 1.03, 95% CI: 1.01–1.06, *p* = 0.011) but not in the more recent eras; E3 (aOR: 1.01, 95% CI: 0.99–1.03, *p* = 0.592); E4 (aOR: 1.00, 95% CI: 0.97–1.03, *p* = 0.926). Additionally, the use of basiliximab/daclizumab was associated with lower odds of DGF compared to the use of alemtuzumab in E3 (aOR: 0.44, 95% CI: 0.24–0.79, *p* = 0.007) and E4 (aOR: 0.19, 95% CI: 0.08–0.43, *p* < 0.001) but not in the earlier eras of E1 (aOR: 1.37, 95% CI: 0.90–2.07, *p* = 0.140) nor E2 (aOR: 0.83, 95% CI: 0.24–2.94, *p* = 0.778) (Table 2).

### 3.2 Overall Risk Factors Without Era Stratification

When analyzing the entire cohort (including all eras) together, recipient BMI (aOR: 1.07, 95% CI: 1.05–1.09, *p* < 0.001) and previous transplant (aOR: 1.37, 95% CI: 1.08–1.74, *p* = 0.010) were shown to be risk factors for DGF (Supplemental Table 2). These findings remained when excluding preemptive transplant: recipient BMI (aOR: 1.07, 95% CI: 1.05–1.09, *p* < 0.001) and previous transplant (aOR: 1.30, 95% CI: 1.02–1.66, *p* = 0.034) (Supplemental Table 3).

In contrast, glomerulonephritis as the cause of recipient ESKD compared to diabetes as the cause of recipient ESKD was associated with a lower odds of DGF (aOR: 0.74, 95% CI: 0.57–0.95, *p* = 0.018) in the E1‐E4 cohort (Supplemental Table 2), which remained the case after excluding preemptive transplant (aOR: 0.74, 95% CI: 0.57–0.96, *p* = 0.022) (Supplemental Table 3). Female recipient sex (aOR: 0.76, 95% CI: 0.63–0.92, *p* = 0.004) and preemptive transplant (aOR: 0.14, 95% CI: 0.09–0.22, *p* < 0.001) were also associated with a lower odds of DGF (Supplemental Table 2). Female recipient sex also remained associated with a lower odds of DGF when excluding preemptive transplant from analysis (aOR: 0.75, 95% CI: 0.62–0.91, *p* = 0.003) (Supplemental Table 3).

Donor age (aOR: 1.20, 95% CI: 1.10–1.30, *p* < 0.001), donor BMI (aOR: 1.01, 95% CI: 1.00–1.03, *p* = 0.018), DCD (aOR: 3.86, 95% CI: 3.17–4.70, *p* < 0.001), terminal serum creatinine (aOR: 1.63, 95% CI: 1.38–1.94, *p* < 0.001), and CIT > 24 h compared to < 12 h (aOR: 1.42, 95% CI: 1.05–1.91, *p* = 0.021) were also shown to be risk factors for DGF throughout the entire study period (Supplemental Table 2). These findings remained after excluding preemptive transplant from analysis: donor age (aOR: 1.21, 95% CI: 1.11–1.32, *p* < 0.001), donor BMI (aOR: 1.02, 95% CI: 1.00–1.03, *p* = 0.011), DCD (aOR: 3.97, 95% CI: 3.24–4.85, *p* < 0.001), terminal serum creatinine (aOR: 1.65, 95% CI: 1.38–1.97, *p* < 0.001), and CIT > 24 h compared to < 12 h (aOR: 1.44, 95% CI: 1.06–1.95, *p* = 0.019) (Supplemental Table 3).

### 3.3 Outcomes

**FIGURE 1:**
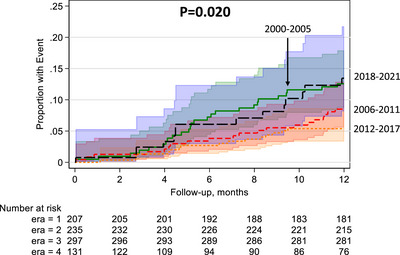
Kaplan–Meier analysis of deceased donor kidney transplant recipients with uncensored graft failure by era.

There was a trend towards decreased risk of first‐year UCGF in the setting of DGF in E2 (uHR: 0.68, 95% CI: 0.43–1.06, *p* = 0.090), E3 (uHR: 0.34, 95% CI: 0.20–0.57, *p* < 0.001), and E4 (uHR: 0.79, 95% CI: 0.46–1.37, *p* = 0.410) compared to E1 (Table 3). Kaplan–Meier analysis at one‐year follow‐up confirmed this (Figure 1). This trend remained when adjusting for baseline characteristics in E2 (aHR: 0.74, 95% CI: 0.35–1.60, *p* = 0.450), E3 (aHR: 0.42, 95% CI: 0.20–0.87, *p* = 0.020), and E4 (aHR: 0.97, 95% CI: 0.44–2.18, *p* = 0.950) in comparison to E1.

**TABLE 3:** Outcomes of deceased donor kidney transplant recipients with DGF.

	Era of DGF			
	2000–2005	2006–2011	2012–2017	2018–2021
**Total graft failure in first year (uncensored)**				
Inc Rate	21.5	14.4	7.1	17.6
Unadjusted HR	Ref	0.68 (0.43, 1.06); 0.090	0.34 (0.20, 0.57); **<0.001**	0.79 (0.46, 1.37); 0.410
Adjusted HR	Ref	0.74 (0.35, 1.60); 0.450	0.42 (0.20, 0.87); **0.020**	0.97 (0.44, 2.18); 0.950
**Death‐censored graft failure in first year**				
Inc Rate	5.67	7.51	2.78	9.87
Unadjusted HR	Ref	1.33 (0.62, 2.83); 0.460	0.49 (0.20, 1.22); 0.130	1.73 (0.73, 4.07); 0.210
Adjusted HR	Ref	1.64 (0.60, 4.48); 0.340	0.52 (0.18, 1.50); 0.230	1.68 (0.59, 4.82); 0.330
**Death with a functioning graft in first year**				
Inc Rate	7.73	1.32	2.77	2.96
Unadjusted HR	Ref	0.17 (0.05, 0.59); **0.005**	0.35 (0.15, 0.83); **0.020**	0.41 (0.12, 1.41); 0.160
Adjusted HR	Ref	0.16 (0.04, 0.71); **0.020**	0.34 (0.12, 0.95); **0.040**	0.47 (0.11, 1.97); 0.300
**Acute rejection in first year**				
Inc Rate	52.2	69.0	20.6	7.14
Unadjusted HR	Ref	1.29 (0.96, 1.75); 0.100	0.43 (0.30, 0.62); **<0.001**	0.13 (0.06, 0.29); **<0.001**
Adjusted HR	Ref	1.32 (0.89, 1.94); 0.170	0.46 (0.30; 0.69); **<0.001**	0.16 (0.07, 0.36); **<0.001**

HR: hazard ratio; Inc: Incidence; DGF: delayed graft function; Ref: Reference.

**FIGURE 2:**
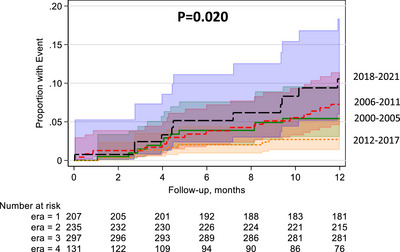
Kaplan–Meier analysis of deceased donor kidney transplant recipients with death‐censored graft failure by era.

There was a numerical decreased risk of first‐year DCGF in the setting of DGF in E3 (uHR: 0.49, 95% CI: 0.20–1.22, *p* = 0.130) and a numerical increased risk of first‐year DCGF in the setting of DGF in E2 (uHR: 1.33, 95% CI: 0.62–2.83, *p* = 0.460) and E4 (uHR: 1.73, 95% CI: 0.73–4.07, *p* = 0.210). Kaplan–Meier analysis at one‐year follow‐up confirms this (Figure 2). These numerical findings were also found after adjusting for baseline characteristics: E2 (aHR: 1.64, 95% CI: 0.60–4.48, *p* = 0.340), E3 (aHR: 0.52, 95% CI: 0.18–1.50, *p* = 0.230), and E4 (aHR: 1.68, 95% CI: 0.59–4.82, *p* = 0.330) compared to E1.

**FIGURE 3:**
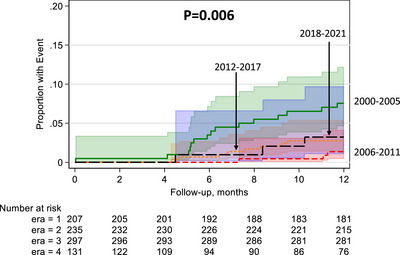
Kaplan–Meier analysis of deceased donor kidney transplant recipients who died with a functioning graft by era.

There was a trend towards decreased risk of first‐year DWFG in the setting of DGF in E2 (uHR: 0.17, 95% CI: 0.05–0.59, *p* = 0.005), E3 (uHR: 0.35, 95% CI: 0.15–0.83, *p* = 0.020), and E4 (uHR: 0.41, 95% CI: 0.12–1.41, *p* = 0.160) compared to E1. This was confirmed by Kaplan–Meier analysis (Figure 3). This trend remained in E2 (aHR: 0.16, 95% CI: 0.04–0.71, *p* = 0.020), E3 (aHR: 0.34, 95% CI: 0.12–0.95, *p* = 0.040), and E4 (aHR: 0.47, 95% CI: 0.11–1.97, *p* = 0.300) compared to E1 when adjusting for baseline characteristics.

**FIGURE 4:**
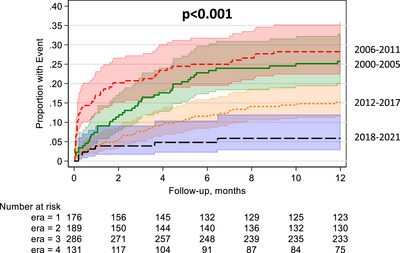
Kaplan–Meier analysis of deceased donor kidney transplant recipients with acute rejection by era.

Additionally, the risk of first‐year AR in the setting of DGF was significantly lower in E3 (uHR: 0.43, 95% CI: 0.30–0.62, *p* < 0.001) and E4 (uHR: 0.13, 95% CI: 0.06–0.29, *p* < 0.001) compared to E1. This was further confirmed by Kaplan–Meier analysis (Figure 4). The same observation was found when adjusting for baseline characteristics in E3 (aHR: 0.46; 95% CI: 0.30–0.69, *p* < 0.001) and E4 (aHR: 0.16; 95% CI: 0.07–0.36, *p* < 0.001).

## 4 Discussion

In this large cohort of 3085 DDKTRs, we identified variation in the risk factors for DGF. In each era, DCD, higher donor terminal serum creatinine, and pretransplant duration of dialysis were risk factors for DGF, while preemptive transplant was associated with a lower incidence of DGF. Other factors, such as higher recipient BMI, higher donor BMI, and induction immunosuppression, were not consistently associated with the incidence of DGF in each era. The median duration of DGF and the median number of dialysis sessions needed were lower in recent eras compared to previous eras. Additionally, among DDKTRs with DGF, we saw a significantly reduced risk of AR in the setting of DGF in both the 2012–2017 and 2018–2021 eras compared to the 2000–2005 era. There was also a trend of improved one‐year uncensored graft survival and patient survival following DGF in the recent eras.

The factors found to be associated with increased risk for DGF in each era of our study were consistent with the findings of other publications. Donor terminal serum creatinine as a risk factor for DGF aligns with its use as an indication of kidney function. Also, in their multivariable logistic regression analysis of DDKTRs in 2003–2006, Irish et al. found that donor terminal serum creatinine levels and the use of kidneys from DCDs were associated with DGF [8]. However, in a study among 2543 DDKTRs at the University of Wisconsin, Zhou et al. showed that DGF was associated with increased risk for AR and graft failure with no significant differences between DCD and DBD [13]. Pretransplant duration of dialysis has also been shown to be a risk factor for DGF in the literature [17, 18, 19].

Although some factors were not consistently associated with increased risk of DGF in each era, overall cohort analysis without era stratification showed some to be risk factors for DGF. For example, recipient BMI was not found to be risk factor in each separate era but was a risk factor for DGF in the overall study period analysis, which aligns with the literature, and could be related to increased operative times and thrombin generation associated with obesity [20, 21, 22]. Additionally, although donor age was not shown to be a risk factor for DGF in each era, overall analysis without era stratification did show it to be a risk factor for DGF as seen in other studies [8].

The reduced risk of DGF with preemptive transplant that we saw in each era and in the overall study period analysis is also well‐supported. In their 2000–2004 analysis of 1585 patients in four French transplant centers, Kessler et al. found that patients who received pretransplant dialysis were more likely to have DGF than patients who had a preemptive transplant [23]. In a 2006–2008 retrospective single‐center study, Luo et al. saw similar findings [24]. However, despite preemptive transplant being a leading treatment for ESKD patients because of improved outcomes, optimal timing for preemptive transplants needs further study [25].

There is conflicting evidence regarding the relationship between the duration of DGF and outcomes. For instance, Shamali et al. did not see an association between the duration of DGF and graft survival in 225 DCD donor kidney transplants from 2011–2016 [26]. In contrast, in their single‐center retrospective study of 1714 DDKTRs from 2008–2020, Budhiraja et al. found that the duration of DGF was associated with death‐censored graft survival only for patients with a duration of DGF greater than 28 days [27]. In a study examining 2006–2016 DCD donors, Phillips et al. saw that a duration of DGF greater than 14 days was associated with an increased risk of DCGF and death relative to non‐DGF patients [28]. They also found that a duration of DGF greater than 14 days was associated with an increased rate of AR within 3 months of transplant compared to a duration of DGF less than 7 days [28]. In our findings, in the eras of decreased risk of one‐year AR in the setting of DGF, 2012–2017 and 2018–2021, there was also a greater proportion with a duration of DGF between 1–6 days than greater or equal to 14 days. Therefore, although our cohort's reduced median duration of DGF in the recent eras could contribute to improved outcomes of DGF, more research is needed given the varying findings in the literature.

DGF is associated with increased rates of AR [9, 29, 30, 31]. The progression of DGF has been linked to ischemia‐reperfusion injury (IRI), occurring during the pre‐procurement, procurement, transport, implantation, and reperfusion stages of kidney transplantation [31, 32, 33]. Prolonged warm ischemia time (WIT) or CIT may lead to hypoxic cells, resulting in limited ATP production, reactive oxidative species generation, and cytoskeletal abnormalities [8, 32, 34, 35]. These effects can lead to cellular and tissue damage. After implantation of the kidney in the recipient, reperfusion may lead to activation of the complement system, increased inflammation and oxidative stress, and further cellular damage and death [31, 32]. During reperfusion, the recipient's innate and adaptive immune responses are also activated [32, 33, 36, 37]. Toll‐like receptors recognize cellular debris as danger‐associated molecular patterns, resulting in inflammation [32, 36, 37]. Recruited dendritic cells present alloantigens to T‐lymphocytes, eventually leading to AR [31, 32].

However, immunologic factors and improvements in management of DGF over time may explain the significantly reduced risk of AR in the DGF setting in our 2012–2017 and 2018–2021 transplant cohorts compared to that of the 2000–2005 era. In the recent eras of 2012–2017 and 2018–2021, there was also a lower proportion of individuals who had received a previous transplant relative to 2000–2005. This could potentially contribute to the decreased risk of AR in the recent eras. However, 2006–2011 also had a lower proportion of individuals who had received a previous transplant compared to 2000–2005, suggesting that this does not play an independent role in this. Although the previous transplant was not shown to be a risk factor for DGF in each era of our study, this could be due to it being underpowered in each era, however, it was shown to be a risk factor when removing era stratification (Supplemental Tables 2 and 3). Interestingly, the use of the nondepleting induction immunosuppression agents basiliximab/daclizumab, was associated with lower odds of DGF in the recent eras compared to the depleting agent, alemtuzumab. This conflict with the Kidney Disease: Improving Global Outcomes (KDIGO) guidelines, in which a lymphocyte‐depleting agent is recommended for patients with immunological risk [38]. This could also be due to the selection bias, as recipients with a high risk of DGF were less likely to receive a depleting agent for induction. Similarly, Ravindra et al. conducted an analysis of 65 848 patients in the United Network for Organ Sharing/Organ Procurement and Transplantation Network (UNOS/OPTN) from 2006–2015. They found that depleting agents were associated with decreasing odds for the composite outcome of one‐year AR and graft failure, especially for those with increased risk of DGF [39].

Clinical practices for DGF could also contribute to the reduced risk of AR in the DGF setting seen in recent eras. In their retrospective observational study of DDKTRs at the University of Wisconsin between July 2009 and July 2014, Muth et al. saw that patients in the outpatient DGF clinic (starting in July 2011) were less likely to develop AR compared to pre‐DGF clinic patients, aligning with our findings. The multidisciplinary DGF clinic with close follow‐up in clinic three times a week likely contributed significantly to this improvement. Additionally, this could be related to varying AR detection thresholds and advancements in immunosuppression [11]. Calcineurin inhibitors (CNIs) have been shown to reduce AR incidence with their role in suppressing T‐cell stimulation [40]. Interestingly, Muth et al. saw significantly lower discharge tacrolimus levels in the post‐DGF clinic group compared to the pre‐DGF clinic group. As CNIs can also lead to the activation of the complement system, increased vasoconstriction, and organ impairment [32, 41], the timing of the immunosuppression therapy components may be important. While it has been proposed that delaying CNI initiation has some benefit [37], Ghadimi et al. saw that thymoglobulin induction and delayed tacrolimus initiation were not associated with lower frequency or duration of DGF but were associated with increased 3‐month graft survival [42].

Although Muth et al. did not see a statistical difference in graft survival nor patient survival in the pre‐DGF clinic versus post‐DGF clinic patients, their Kaplan–Meier analysis shows that at one‐year follow‐up, pre‐DGF clinic patients had a higher numerical incidence of death [11]. In our present study, we similarly saw a numerical trend in reduced risk of one‐year DWFG in the recent eras. The reduced numerical risk of DCGF we saw in 2012–2017 could potentially be related to the findings of AR as a partial mediator between duration of DGF and subsequent death‐censored graft survival [28]. However, we did not see this same trend of reduced risk of DCGF in 2018–2021, when the risk for AR was also significantly reduced. This mediation relationship has not been consistent and therefore warrants further study [43].

This single‐center observational study design has inherent limitations, preventing us from reporting causation within our findings. As this study is focused on the University of Wisconsin patients and procedures, there is limited generalizability of our findings to other populations and practices of other health systems. Additionally, as documentation practices of dialysis services varied over the study period and amongst clinicians, duration of DGF and dialysis count were estimated using standard guidelines as needed. Although all kidney allografts were preserved with University of Wisconsin solution, data on pump parameters, kidney imports or local were not consistently collected in our database and therefore not included in this study. There were changes in the machine perfusion practice during the study period. It was utilized earlier, but since 2011–2020, its utilization declined, mainly based on the organ accepting surgeon's discretion, considering availability of the operating room, risk of DGF, and other factors. However, we have been utilizing machine perfusion regularly again. Moreover, there likely could have been other variable donor procurement and kidney storage practices that were not assessed in this study. However, the large sample size and 22‐year length of this study capture a large data set and shed light on the variances in risk factors for and outcomes of DGF since 2000, contributing to an area of limited research.

In conclusion, this study showed that DCD, higher donor terminal serum creatinine, and pretransplant duration of dialysis remained risk factors for DGF, while preemptive transplant remained associated with lower incidence of DGF in each era of our study. We showed a statistical improvement in the risk of AR in the setting of DGF in recent eras, likely due to immunologic factors and improved management of DGF, particularly with a multidisciplinary DGF clinic with close follow‐up. Our findings also support a trend in improved uncensored graft and patient survival in recent eras. Such findings are important for proper donor and recipient selection, along with prevention and treatment of DGF. However, further studies are needed to more precisely evaluate changes in immunological, clinical, and pharmacological practices over time and their potential impacts on patients with DGF and related outcomes.

## Author Contributions

Camille C. Ylagan: Manuscript preparation, design, data collection, writing. Paul E. Schindler: Editing, data collection. Dave B. Patel: Editing, data collection. Carrie Thiessen: Editing, analysis. Adam P. Bregman: Editing. Didier Mandelbrot: Editing. Brad C. Astor: Statistical analysis, data collection, editing. Sandesh Parajuli: Original idea, data collection, writing, editing.

## Funding

This study was supported by Dr. Mandelbrot has unrestricted research funds from the Virginia Lee Cook Foundation. The data that supports the findings of this study are available from the corresponding author, [SP], upon reasonable request.

## Disclosure

The authors have nothing to report.

## Conflicts of Interest

The author does not declare any conflict of interest.

## Supporting information




**Supplemental Table 1**. Risk factors for DGF for deceased donor kidney transplant patients by era, excluding preemptive transplants.

**Supplemental Table 2**. Risk factors for DGF for deceased donor kidney transplant patients during the entire study period (2000‐2021).

**Supplemental Table 3**. Risk factors for DGF for deceased donor kidney transplant patients during the entire study period (2000‐2021), excluding preemptive transplant.

## Data Availability

The data that support the findings of this study are available from the corresponding author upon reasonable request.
