# Supplementary material for: Changes by Era in Risk Factors and Outcomes Among Deceased Donor Kidney Transplant Recipients With Delayed Graft Function
Source: Clin Transplant. 2026 Feb 15;40(2):e70484. doi: 10.1111/ctr.70484 (PMC12906863; doi:10.1111/ctr.70484)
Supplement: Supplementary file 1 — Supplemental Table 1. Risk factors for DGF for deceased donor kidney transplant patients by era, excluding preemptive transplants. [file CTR-40-e70484-s003.docx]

**Supplemental Table 1.** Risk factors for DGF for deceased donor kidney transplant patients by era, excluding pre-emptive transplants.

|  | | **2000-2005** | **2006-2011** | **2012-2017** | **2018-2021** |
| --- | --- | --- | --- | --- | --- |
|  | | **aOR (95% CI; P)** | **aOR (95% CI; P)** | **aOR (95% CI; P)** | **aOR (95% CI; P)** |
| Recipients  Factors | Age (per year) | 0.94 (0.79, 1.12), 0.494 | 0.89 (0.76, 1.05), 0.167 | 0.99 (0.85, 1.14), 0.856 | 1.23 (0.98, 1.55), 0.079 |
|  | Female | 0.75 (0.51, 1.12), 0.160 | 0.79 (0.54, 1.14), 0.204 | 0.71 (0.50, 1.02), 0.062 | 0.56 (0.33, 0.94), **0.029** |
|  | Non-White | 1.37 (0.85, 2.23), 0.198 | 1.91 (1.25, 2.92), **0.003** | 0.84 (0.57, 1.23), 0.363 | 1.12 (0.65, 1.95), 0.679 |
|  | Body Mass Index (per Kg/m2) | 1.08 (1.05, 1.12), **<0.001** | 1.09 (1.05, 1.13), **<0.001** | 1.06 (1.02, 1.09), **0.001** | 1.04 (0.99, 1.10), 0.093 |
|  | Causes of ESKD (%)  Diabetes  Hypertension  Glomerulonephritis  Polycystic Kidney Disease  Other | Ref  0.54 (0.29, 1.00), 0.051  0.56 (0.33, 0.95), **0.031**  0.55 (0.27, 1.12), 0.100  0.68 (0.40, 1.13), 0.135 | Ref  0.78 (0.44, 1.41), 0.416  0.67 (0.41, 1.10), 0.110  0.71 (0.38, 1.31), 0.269  0.88 (0.52, 1.47), 0.615 | Ref  1.03 (0.62, 1.71), 0.901  0.96 (0.59, 1.56), 0.864  0.49 (0.23, 1.02), 0.057  0.97 (0.59, 1.61), 0.907 | Ref  1.15 (0.58, 2.30), 0.686  0.71 (0.35, 1.48), 0.363  0.86 (0.36, 2.01), 0.720  0.91 (0.43, 1.94), 0.806 |
|  | Induction Immunosuppression  Alemtuzumab  Anti-thymocyte Globulin  Basiliximab/Daclizumab | Ref  1.68 (0.92, 3.08), 0.093  1.31 (0.86, 2.01), 0.212 | Ref  1.19 (0.32, 4.42), 0.795  0.82 (0.23, 2.93), 0.754 | Ref  0.54 (0.29, 1.03), 0.062  0.41 (0.22, 0.76), **0.005** | Ref  0.36 (0.19, 0.69), **0.002**  0.17 (0.07, 0.39), **<0.001** |
| Immunologic  Factors | HLA Mismatch (per 1) | 1.03 (0.92, 1.15), 0.646 | 0.98 (0.87, 1.10), 0.721 | 1.08 (0.96, 1.21), 0.225 | 0.88 (0.74, 1.05), 0.163 |
|  | Previous Transplant | 1.45 (0.87, 2.41), 0.155 | 0.92 (0.56, 1.49), 0.724 | 1.3 (0.82, 2.08), 0.263 | 1.28 (0.62, 2.66), 0.499 |
| Donor  Factors | Age (per year) | 1.11 (0.93, 1.32), 0.238 | 1.32 (1.09, 1.59), **0.004** | 1.24 (1.06, 1.45), **0.007** | 1.17 (0.92, 1.48), 0.211 |
|  | Female | 1.13 (0.77, 1.65), 0.544 | 0.88 (0.59, 1.29), 0.500 | 0.95 (0.66, 1.36), 0.782 | 0.58 (0.34, 0.98), **0.043** |
|  | Non-White | 0.87 (0.42, 1.79), 0.707 | 1.59 (0.74, 3.38), 0.232 | 0.68 (0.35, 1.35), 0.273 | 1.04 (0.46, 2.37), 0.920 |
|  | Body Mass Index (per Kg/m2) | 1.04 (1.01, 1.07), **0.003** | 1.03 (1.01, 1.06), **0.010** | 1.01 (0.99, 1.03), 0.402 | 0.99 (0.96, 1.02), 0.542 |
|  | Cause of Death:  Cardiovascular | 1.33 (0.87, 2.04), 0.186 | 1.45 (0.96, 2.18), 0.080 | 0.91 (0.60, 1.37), 0.636 | 1.14 (0.63, 2.06), 0.668 |
|  | DCD | 3.80 (2.42, 5.98), **<0.001** | 4.38 (2.94, 6.55), **<0.001** | 5.37 (3.71, 7.78), **<0.001** | 2.87 (1.70, 4.86), **<0.001** |
|  | Terminal Serum Creatinine (mg/dl) | 1.22 (1.01, 1.46), **0.035** | 1.84 (1.30, 2.61), **0.001** | 2.58 (1.76, 3.77), **<0.001** | 1.91 (1.26, 2.90), **0.002** |
|  | Kidney Donor Profile Index | 1.00 (0.99, 1.01), 0.610 | 1.00 (0.99, 1.01), 0.964 | 1.01 (1.00, 1.02), 0.115 | 1.01 (1.00, 1.03), 0.167 |
|  | Right Kidney | 1.01 (0.70, 1.47), 0.957 | 1.20 (0.83, 1.72), 0.329 | 1.57 (1.13, 2.20), **0.008** | 1.44 (0.89, 2.34), 0.140 |
|  | Cold Ischemia Time  <12 hours  12-18 hours  19-24 hours  >24 hours | Ref  1.33 (0.54, 3.29), 0.535  1.59 (0.67, 3.80), 0.294  2.11 (0.86, 5.17), 0.104 | Ref  1.03 (0.69, 1.56), 0.869  1.48 (0.89, 2.46), 0.129  0.94 (0.41, 2.16), 0.892 | Ref  0.96 (0.63, 1.47), 0.847  1.14 (0.73, 1.77), 0.563  1.48 (0.81, 2.71), 0.207 | Ref  1.02 (0.57, 1.82), 0.940  0.94 (0.50, 1.79), 0.856  1.20 (0.51, 2.86), 0.676 |

*All variables were used for adjusted models.

aOR: adjusted odds ratio; CI: confidence interval; cPRA: calculated panel reactive antibody

DCD: donation after circulatory death; DGF: delayed graft function; ESKD: end-stage kidney disease

HLA: human leukocyte antigen; Ref: reference
