# Supplementary material for: Changes by Era in Risk Factors and Outcomes Among Deceased Donor Kidney Transplant Recipients With Delayed Graft Function
Source: Clin Transplant. 2026 Feb 15;40(2):e70484. doi: 10.1111/ctr.70484 (PMC12906863; doi:10.1111/ctr.70484)
Supplement: Supplementary file 2 — Supplemental Table 2. Risk factors for DGF for deceased donor kidney transplant patients during the entire study period (2000‐2021). [file CTR-40-e70484-s001.docx]

**Supplemental Table 2.** Risk factors for DGF for deceased donor kidney transplant patients during the entire study period (2000-2021).

|  | | **2000-2021** |
| --- | --- | --- |
| **Variables** | | **aOR (95% CI; P)** |
| Recipients  Factors | Age (per year) | 0.95 (0.88, 1.03), 0.189 |
|  | Female | 0.76 (0.63, 0.92), **0.004** |
|  | Non-White | 1.22 (0.99, 1.51), 0.063 |
|  | Body Mass Index (per Kg/m2) | 1.07 (1.05, 1.09), **<0.001** |
|  | Causes of ESKD (%)  Diabetes  Hypertension  Glomerulonephritis  Polycystic Kidney Disease  Other | Ref  0.86 (0.65, 1.14), 0.298  0.74 (0.57, 0.95), **0.018**  0.73 (0.53, 1.01), 0.061  0.84 (0.65, 1.08), 0.176 |
|  | Induction Immunosuppression  Alemtuzumab  Anti-thymocyte Globulin  Basiliximab/Daclizumab | Ref  1.06 (0.82, 1.38), 0.652  0.91 (0.71, 1.15), 0.424 |
|  | Pre-emptive Transplant | 0.14 (0.09, 0.22), **<0.001** |
| Immunologic  Factors | HLA Mismatch (per 1) | 1.01 (0.95, 1.07), 0.704 |
|  | Previous Transplant | 1.37 (1.08, 1.74), **0.010** |
| Donor  Factors | Age (per year) | 1.20 (1.10, 1.30), **<0.001** |
|  | Female | 0.88 (0.73, 1.07), 0.201 |
|  | Non-White | 0.81 (0.58, 1.15), 0.248 |
|  | Body Mass Index (per Kg/m2) | 1.01 (1.00, 1.03), **0.018** |
|  | Cause of Death:  Cardiovascular | 1.17 (0.95, 1.44), 0.139 |
|  | DCD | 3.86 (3.17, 4.70), **<0.001** |
|  | Terminal Serum Creatinine (mg/dl) | 1.63 (1.38, 1.94), **<0.001** |
|  | Kidney Donor Profile Index | 1.00 (1.00, 1.01), 0.093 |
|  | Right Kidney | 1.18 (0.99, 1.41), 0.070 |
|  | Cold Ischemia Time  <12 hours  12-18 hours  19-24 hours  >24 hours | Ref  0.95 (0.75, 1.20), 0.673  1.17 (0.92, 1.49), 0.202  1.42 (1.05, 1.91), **0.021** |

*All variables were used for the adjusted model.

aOR: adjusted odds ratio; CI: confidence interval; cPRA: calculated panel reactive antibody

DCD: donation after circulatory death; DGF: delayed graft function; ESKD: end-stage kidney disease

HLA: human leukocyte antigen; Ref: reference
