# Supplementary material for: Changes by Era in Risk Factors and Outcomes Among Deceased Donor Kidney Transplant Recipients With Delayed Graft Function
Source: Clin Transplant. 2026 Feb 15;40(2):e70484. doi: 10.1111/ctr.70484 (PMC12906863; doi:10.1111/ctr.70484)
Supplement: Supplementary file 3 — Supplemental Table 3. Risk factors for DGF for deceased donor kidney transplant patients during the entire study period (2000‐2021), excluding preemptive transplant. [file CTR-40-e70484-s002.docx]

**Supplemental Table 3.** Risk factors for DGF for deceased donor kidney transplant patients during the entire study period (2000-2021), excluding pre-emptive transplant.

|  | | **2000-2021** |
| --- | --- | --- |
| **Variable** | | **aOR (95% CI; P)** |
| Recipients  Factors | Age (per year) | 0.95 (0.88, 1.03), 0.221 |
|  | Female | 0.75 (0.62, 0.91), **0.003** |
|  | Non-White | 1.20 (0.97, 1.48), 0.100 |
|  | Body Mass Index (per Kg/m2) | 1.07 (1.05, 1.09), **<0.001** |
|  | Causes of ESKD (%)  Diabetes  Hypertension  Glomerulonephritis  Polycystic Kidney Disease  Other | Ref  0.88 (0.66, 1.16), 0.361  0.74 (0.57, 0.96), **0.022**  0.65 (0.46, 0.91), **0.013**  0.86 (0.66, 1.12), 0.256 |
|  | Induction Immunosuppression  Alemtuzumab  Anti-thymocyte Globulin  Basiliximab/Daclizumab | Ref  1.02 (0.78, 1.33), 0.908  0.88 (0.69, 1.13), 0.317 |
| Immunologic  Factors | HLA Mismatch (per 1) | 1.00 (0.94, 1.07), 0.921 |
|  | Previous Transplant | 1.30 (1.02, 1.66), **0.034** |
| Donor  Factors | Age (per year) | 1.21 (1.11, 1.32), **<0.001** |
|  | Female | 0.89 (0.74, 1.08), 0.240 |
|  | Non-White | 0.86 (0.60, 1.22), 0.385 |
|  | Body Mass Index (per Kg/m2) | 1.02 (1.00, 1.03), **0.011** |
|  | Cause of Death:  Cardiovascular | 1.16 (0.94, 1.44), 0.170 |
|  | DCD | 3.97 (3.24, 4.85), **<0.001** |
|  | Terminal Serum Creatinine (mg/dl) | 1.65 (1.38, 1.97), **<0.001** |
|  | Kidney Donor Profile Index | 1.00 (1.00, 1.01), 0.165 |
|  | Right Kidney | 1.24 (1.03, 1.48), **0.021** |
|  | Cold Ischemia Time  <12 hours  12-18 hours  19-24 hours  >24 hours | Ref  1.00 (0.79, 1.27), 0.997  1.20 (0.94, 1.54), 0.147  1.44 (1.06, 1.95), **0.019** |

*All variables were used for the adjusted model.

aOR: adjusted odds ratio; CI: confidence interval; cPRA: calculated panel reactive antibody

DCD: donation after circulatory death; DGF: delayed graft function; ESKD: end-stage kidney disease

HLA: human leukocyte antigen; Ref: reference
